# Evaluating International Prostate Symptom Score (IPSS) in Accuracy for Predicting Post-Operative Urinary Retention After Elective Cataract Surgery: A Prospective Study

**DOI:** 10.5539/gjhs.v7n7p93

**Published:** 2015-03-26

**Authors:** Faramarz Fazeli, Shahram Gooran, Majidreza Erfanian Taghvaei, Kimia Fazeli

**Affiliations:** 1 School of medicine, Zahedan University of Medical Sciences, Zahedan, Iran; 2 School of medicine, Tehran University of Medical Sciences, Tehran, Iran; 3 School of medicine, Mashhad University of Medical Sciences, Mashhad, Iran

**Keywords:** post-operative urinary retention, IPSS, anesthesia

## Abstract

**Background:**

Postoperative urinary retention-a common and important complication of surgical procedures, can occur after any form of surgical intervention, in both sexes and all ages regardless of patients’ previous history of urinary problems. The importance of post operative urinary tract retention is due to its effect on development of post operative urinary infection, patient anxiety and discomfort, prolongation of hospital stay and increase in hospital costs and morbidity. The International Prostate Symptom Score (IPSS) is an easy method for quantifying and estimating the association between pre-operative bladder-outflow problems and post-operative urinary retention. The aim of present study was to investigate whether the IPSS could predict the likelihood of patients developing urinary retention after elective cataract surgery.

**Methods:**

One hundred and fourteen male patients older than fifty years old, who were candidate for elective cataract surgery, were enrolled in this study. All patients completed an IPSS questionnaire form before operation, and classified into three groups regarding their score (0-7: mild, 8-19: moderate, 20- 35: severe).

**Results:**

Totally 8 patients (7%) developed post-operative urinary retention during first 24 hours after operation. Of the 8 urinary retention patients, 2 had moderate symptoms and 6had severe symptoms. There was a significant difference in developing postoperative urinary retention between patients having mild symptoms and patients having severe symptoms (P-value: 0.025).

**Conclusion:**

It is concluded that while some litterateurs definitely support the idea that IPSS may be useful for predicting post operative urinary retention, there are still some controversies. Considering our results, it seems that IPSS score is not useful in the accurate prediction of those patients who are likely to develop postoperative retention after surgical procedures other than arthroplasty, and more precise studies are need to be conducted about urinary retention occurring postoperatively in different type surgeries, different methods of anesthesia considering age and gender of patients.

## 1. Introduction

Postoperative urinary retention-a common and important complication of surgical procedures (Jensen et al., 2002; Burger et al., 1997), with incidence of 10-70 (Hozack et al., 1998; Waterhouse et al., 1993) can occur after any form of surgical intervention, in both sexes and all ages regardless of patients’ previous history of urinary problems (Darrah et al., 2009; Tammela et al., 1986). Numerous factors are considered to influence development of urinary retention, including advanced age, immobility, medication used for pain management, route and length of medication administration, increased intravenous fluid intake and type of anesthesia (Burger et al., 1997; Skelly et al., 1992; Williams et al., 1993).

The importance of post operative urinary tract retention is due to its effect on development of post operative urinary infection (Jensen et al., 2002; Burger et al., 1997), patient anxiety and discomfort, prolongation of hospital stay and increase in hospital costs and morbidity (Williams et al., 1993; Petros et al., 1992; Smith et al., 1996). Therefore detection of patients at risk for developing post operative urinary retention is of great importance and may help physicians to take preventative measures in order to avoid it (Cronin et al., 2007).

The International Prostate Symptom Score (Prestil et al., 2008) - a self administered questionnaire, developed and validated by the American Urological Association (AUA)- is an easy method for quantifying lower urinary tract obstruction score pre operatively (Cronin et al., 2007; Sarasin et al., 2007) and estimating the association between pre-operative bladder-outflow problems and post-operative urinary retention (Cronin et al., 2007).

While most of the literature have discussed about validity of IPSS in predicting urinary tract retention after arthroplasty, in this study we aimed to investigate whether the IPSS could predict the likelihood of patients developing urinary retention after elective cataract surgery.

## 2. Material and Methods

One hundred and fourteen male patients older than fifty years old who were admitted to the Alzahra hospital (related to Zahedan university of medical science, Sistan Baluchistan, Iran), who were candidates for elective cataract surgery were enrolled in this prospective analytic-descriptive study. The study was approved by the University Hospital Ethical Committee.

Patient with history of neurologic disorders, urinary tract infection, previous catheterization, urolithiasis and urethral stenosis were excluded from the study.

All patients completed an IPSS questionnaire form before operation, and classified into three groups regarding their score (0-7: mild, 8-19: moderate, 20- 35: severe).

Similar mode of anesthesia was performed among all patients (anesthesia protocol: Midazolam hydrochloride, Atracurium, Lidocaine, Neostigmine, Remifentanil) and all patients received equal amounts of intravenous crystalloids (5ml/kg) prior to operation.

All patients were monitored during first forty eight hour post-operatively for the development of acute urinary retention (defined as need for catheterization).

## 3. Result

Our study group of 114 male patients aged over 50 years- were classified into 3 groups of 38 patients, regarding their IPSS score (mild, moderate and severe with IPSS of (0-7), (8 -19) and (20-35), respectively).

Totally 8 patients (7%) developed post-operative urinary retention during first 24 hours after operation. Of the 8 urinary retention patients, 2 had moderate symptoms and 6had severe symptoms.

Statistical analysis via Fisher’s exact test demonstrated a significant difference in developing postoperative urinary retention between patients having mild symptoms and patients having severe symptoms (P-value: 0.025), while no significant difference was detected among other groups.

**Figure 1:**
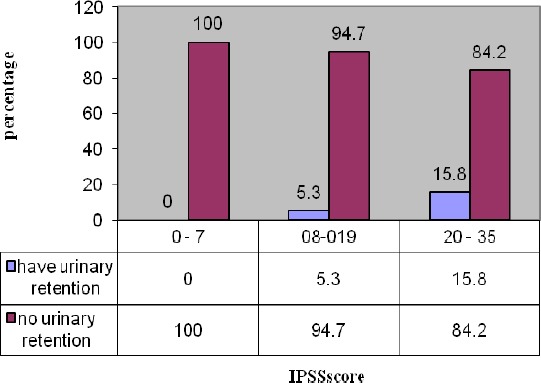
Frequency of post operativeurinary retention regarding IPSS Score 3 groups of mild (0-7), moderate (8-19) and severe (20-35)

## 4. Discussion

7% of patients in our study developed post operative urinary retention which is low compared to previous studies, since rates as low as 10%, and as high as 60% have been reported in the literature (Hozack et al.,1998; Waterhouse et al.,1993). Although in our study IPSS scores seemed to be higher in patients developing urinary retention postoperatively, this correlation is not statistically significant except among mild and severe groups (P=0.025).

In a study performed by J. J. Cronin et al, 118 patients (28 for knee arthroplasty and 90 for hip arthroplasty) were enrolled and the IPSS questionnaire was completed for patients preoperatively. Forty-five patients developed urinary retention post-operatively and the mean IPSS score was significantly higher in patients requiring catheterization. These results show that IPSS could be used to predict the development of postoperative urinary retention in patients presenting for hip or knee arthroplasty (Cronin et al., 2007).

In another study carried out by Elkhodair S et al, a greater chance of developing urinary retention (about 50-100%) after major joint arthroplasty was depicted for patients having moderate to severe score on IPSS (Elkhodair et al., 2006).

On the other hand, a prospective study performed by Sarasin et al, investigated 182 patients undergoing lower limb arthroplasty under spinal anesthesia in order to explain whether postoperative urinary retention can be predicted pre-operatively using the IPSS. Sixty nine percent of males and thirty nine percent of females required catheterization postoperatively. Following logistic regression analysis there was 0.85 predicted probability that males over 70 years would require catheterization. The IPSS score was not useful in predicting retention in either sex at any age (Sarasin et al., 2007).

## 5. Conclusion

It is concluded that while some literateurs definitely support the idea that IPSS may be useful for predicting post operative urinary retention, there are still some controversies.

Considering our results, it seems that IPSS score is not useful in the accurate prediction of those patients who are likely to develop postoperative retention after surgical procedures other than arthroplasty, and more precise studies are need to be conducted about urinary retention occurring postoperatively in different types of surgeries, different methods of anesthesia considering age and gender of patients.
